# Supplementary material for: Integrated molecular diode as 10 MHz half-wave rectifier based on an organic nanostructure heterojunction
Source: Nat Commun. 2020 Jul 17;11:3592. doi: 10.1038/s41467-020-17352-9 (PMC7368027; doi:10.1038/s41467-020-17352-9)
Supplement: Supplementary file 1 — Supplementary Information [file 41467_2020_17352_MOESM1_ESM.pdf]

Supplementary Information

Integrated Molecular Diode as 10 MHz Half-Wave Rectifier Based on an Organic Nanostructure Heterojunction

Tianming Li[1,2,3‡], Vineeth Kumar Bandari[1,2,3‡], Martin Hantusch[4], Jianhui Xin[5], Robert Kuhrt[4], Rachappa Ravishankar[1,2], Longqian Xu[1,2], Jidong Zhang[5], Martin Knupfer[4], Feng Zhu[1,2,3,5]*, Donghang Yan[5], Oliver G. Schmidt[1,2,3]

[1]Material Systems for Nanoelectronics, Chemnitz University of Technology, 09107 Chemnitz, Germany

[2]Institute for Integrative Nanosciences, Leibniz IFW Dresden, 01069 Dresden, Germany

[3]Center for Materials, Architectures and Integration of Nanomembranes (MAIN), Chemnitz University of Technology, 09126 Chemnitz, Germany

[4]Institute for Solid State Research, Leibniz IFW Dresden, 01069 Dresden, Germany

[5]State Key Laboratory of Polymer Physics and Chemistry, Changchun Institute of Applied Chemistry, Chinese Academy of Sciences, 130022 Changchun, China

‡These authors contributed equally to this work.

*e-mail: f.zhu@ifw-dresden.de (current eamil address: zhufeng@ciac.ac.cn)

Supplementary Figures

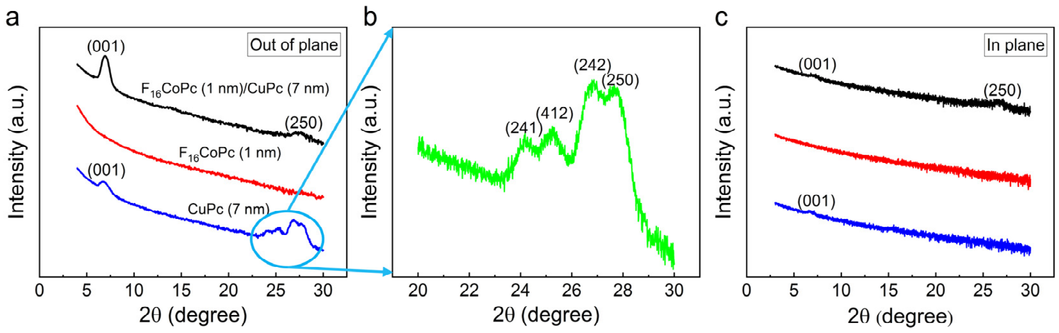

Supplementary Figure 1. XRD of the phthalocyanine molecule layers. a-b, Out of plane XRD and the corresponding magnification of 7 nm CuPc grown on bare Au. **c**, In plane XRD of the corresponding three systems shown in **Figure 1a**.

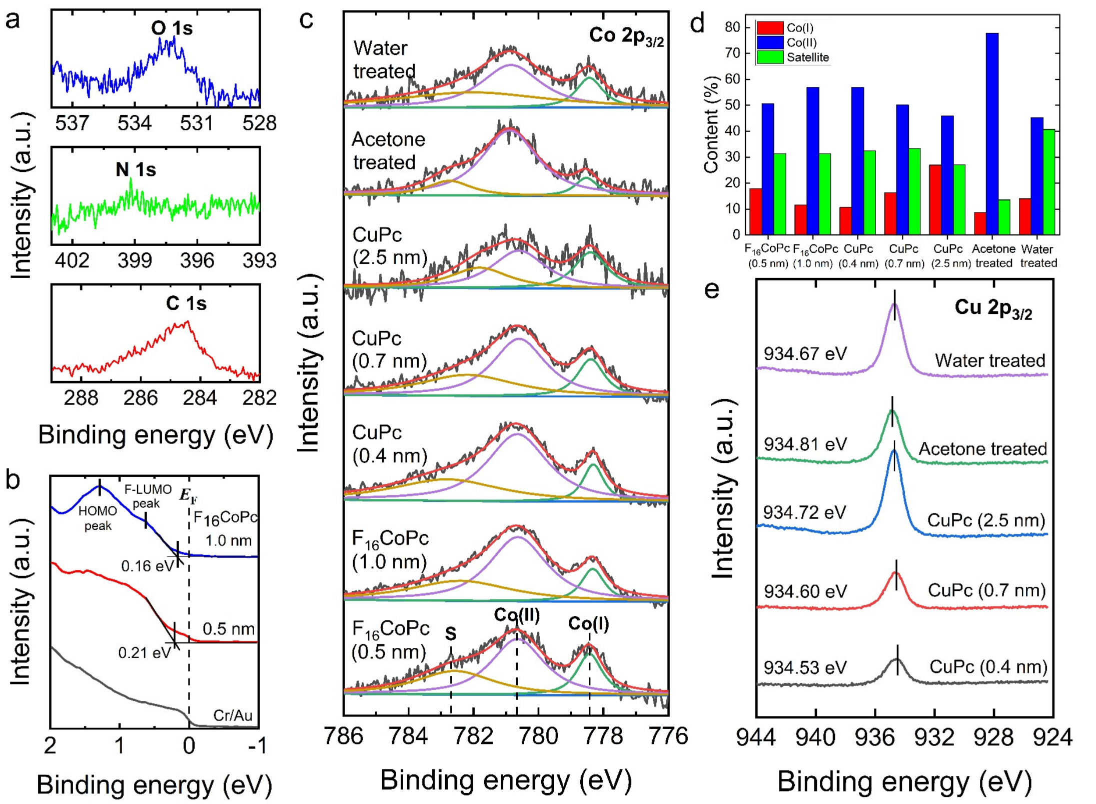

Supplementary Figure 2. UPS&XPS characterizations of phthalocyanine hybrid layers. **a**, XPS spectra (Al-Kα = 1486.6 eV) corresponding O 1s, N 1s, and C 1s core levels of Cr/Au substrate. **b**, Calculated HOMO of F_{16}CoPc layer on Cr/Au substrate by the commonly used linear extrapolation. **c**, XPS spectra of Co $2p_{3/2}$ core levels of the Au/F_{16}CoPc/CuPc system during each step. **d**, Relative contents of Co(I), Co(II) and satellite peak during each step, extracted from **c**. **e**, XPS spectra of Cu $2p_{3/2}$ core levels.

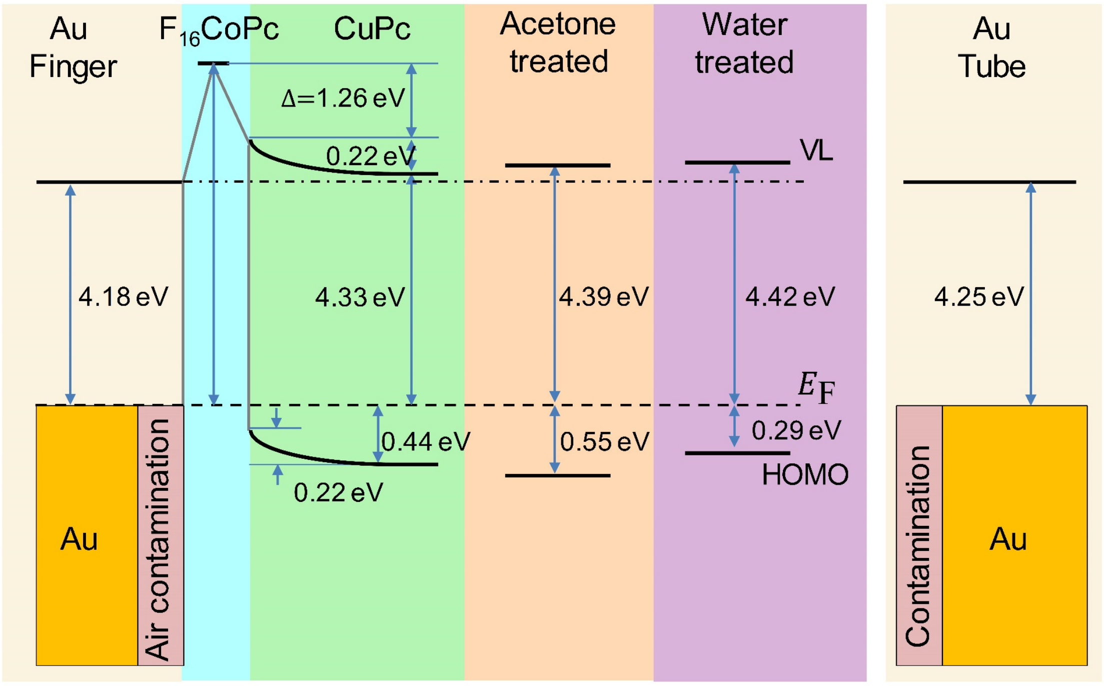

Supplementary Figure 3. Proposed band alignment diagram for the Au (finger)/F_{16}CoPc /CuPc/Au (tube) system.

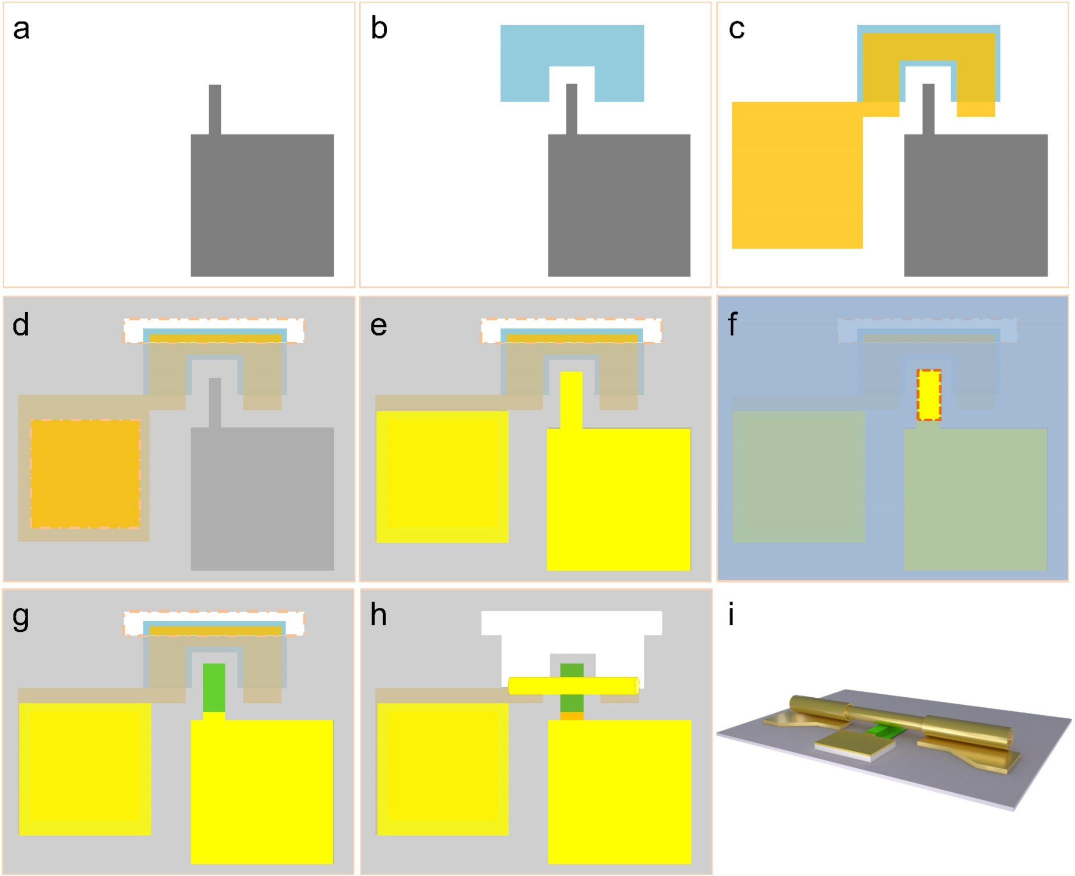

Supplementary Figure 4. Fabrication process of the ultrathin organic diode based on rolled-up soft-contact. **a**, Etching finger mesa by HF solution. **b**, Sacrificial layer Ge. **c**, Strain layers Au/Ti/Cr. **d**, Passivation layer Al_2O_3 with opened windows. **e**, Pad layers Cr/Au. **f**, Mask layer MoO_3 for organic layer. **g**, Organic layer. **h**, Rolling in water. **i**, Sketch of the rolled-up device.

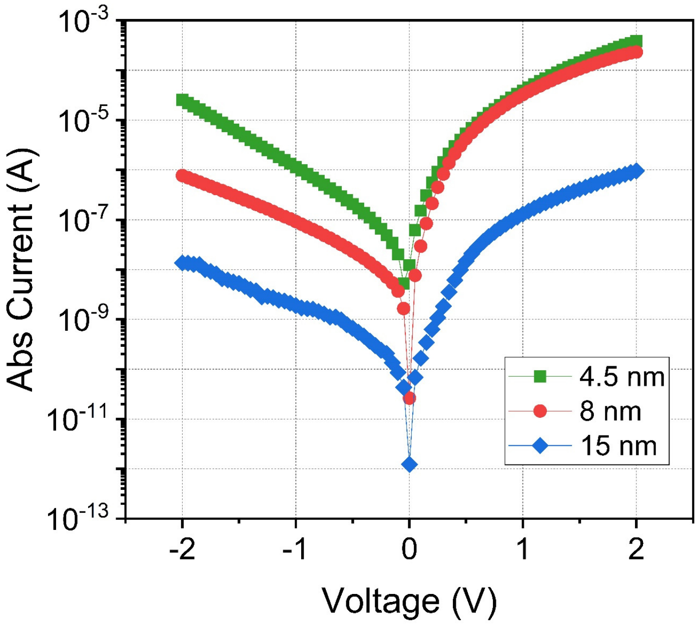

Supplementary Figure 5. ***I-V*** **performance depends on the thickness of the hybrid layer.**

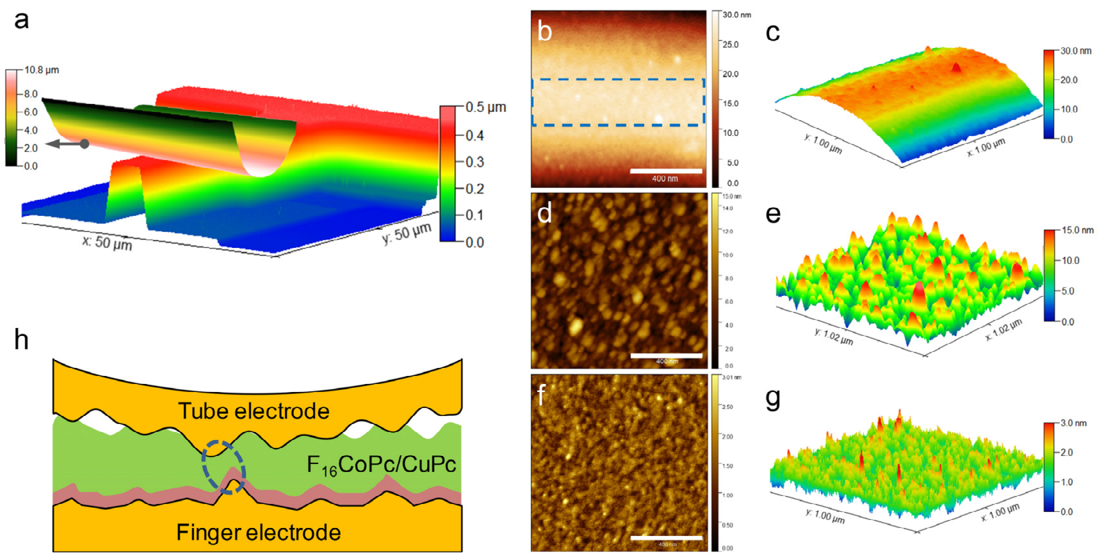

Supplementary Figure 6. Details of the Au (finger)/F_{16}CoPc/CuPc/Au (tube) junction. a, Rolled-up soft-contact demonstrated by AFM image. **b-g**, AFM topography characteristics for rolled-up Au tube (**b** and **c**, measured from the top surface of the tube), Au finger/F_{16}CoPc/CuPc (**d** and **e**), and Au finger (**f** and **g**), respectively. Scale bars, 400 nm. The roughness of the Au tube is extracted from the selected area (in the blue dashed box) along the lateral direction of the tube. **h**, Schematic illustration of the local contacts. The effective gap between the two electrodes shrinks due to the peaks of the Au finger and the tube, as indicated by the blue dashed circle.

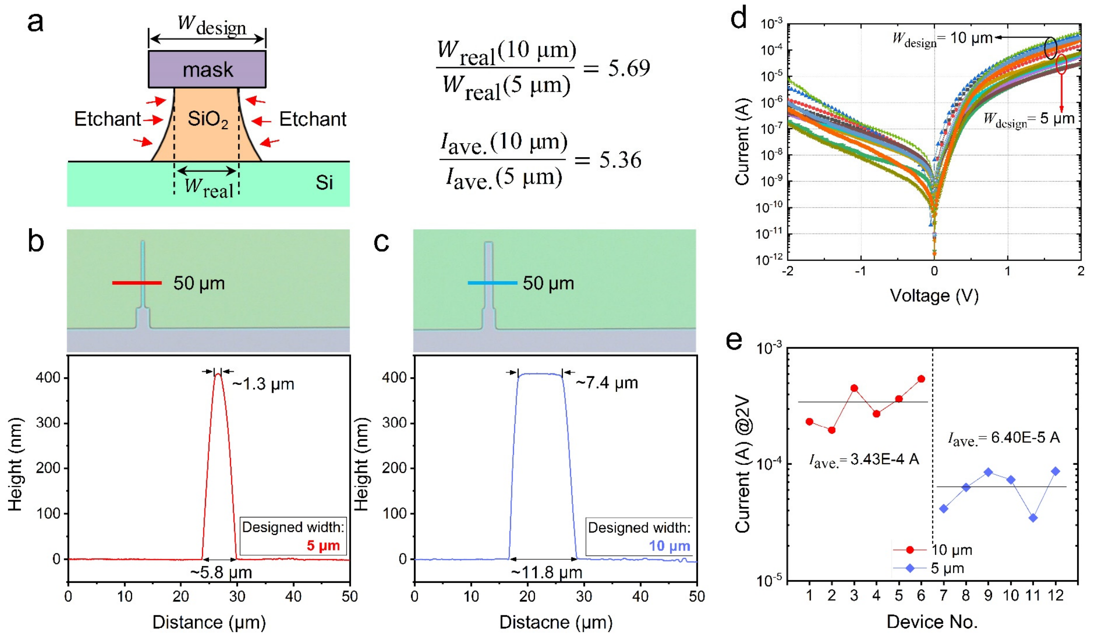

Supplementary Figure 7. Relation of current and mesa width. **a**, Schematic of isotropic under-etching of mesa by HF solution. **b** and **c**, scan (upper panels, microscope images) and the corresponding height profiles (lower panels, obtained by height profile meter) of mesas with $W_{\text{design}} = 5$ and 10 μm. **d**, I-V characteristics of different diodes based on Au (finger)/F16CoPc (1nm)/CuPc (7 nm)/Au (tube). **e**, Average currents (at 2 V) of devices based on $W_{\text{design}} = 5$ and 10 μm.

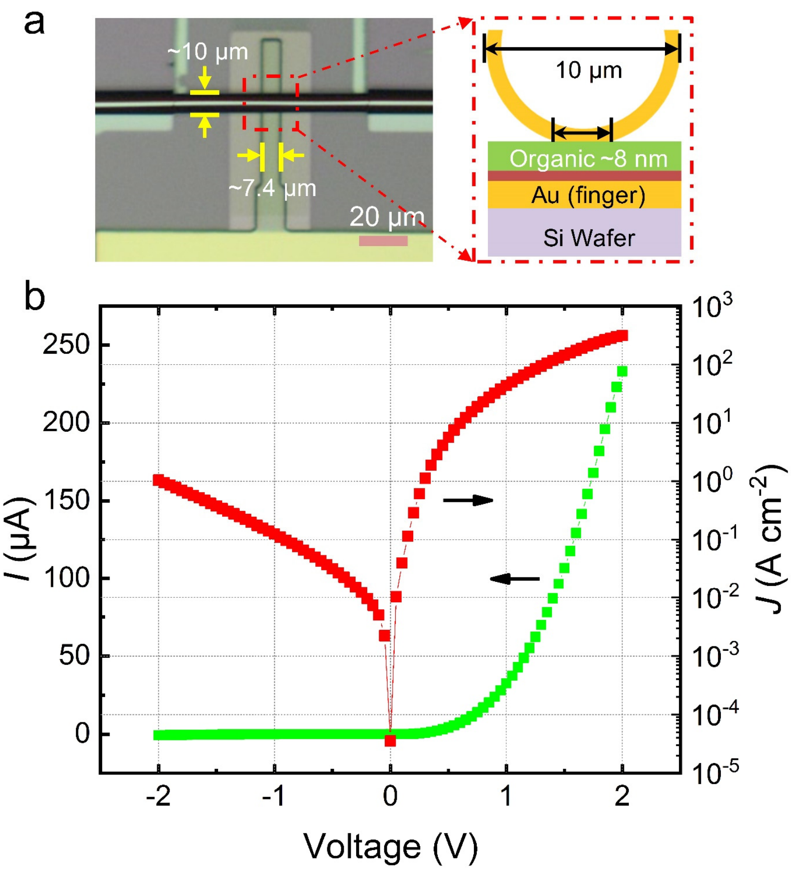

Supplementary Figure 8. Rough estimation of current density of Au (finger)/F_{16}CoPc (1nm)/CuPc (7 nm)/Au (tube), with $W_{design} = 10$ μm. a, The contact area was roughly estimated to be ~74 μm^2 when the designed mesa width is 10 μm (Real contact area should be much less than this value, as depicted in the right panel). **b**, The corresponding current density-voltage (I-V) characteristics.

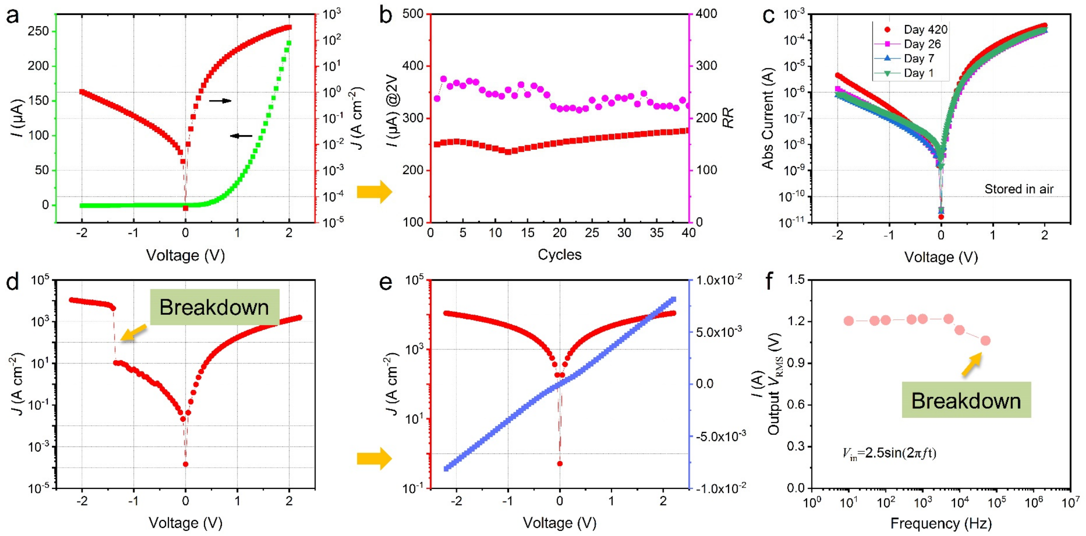

Supplementary Figure 9. Stability of the devices based on Au (finger)/F_{16}CoPc (1nm)/CuPc (7 nm)/Au (tube), with W_{design} = 10 μm. a, Typical I-V characteristics. **b**, corresponding device cycling performance: cycling number dependent current at 2V and rectification ratio (RR). **c**, I-V characteristics over 420 days. **d**, Device breakdown during the I-V measurement caused by Joule heating. **e**, I-V characteristics of the device in **d** after breakdown. **f**, Demonstration of device breakdown during the frequency measurement.

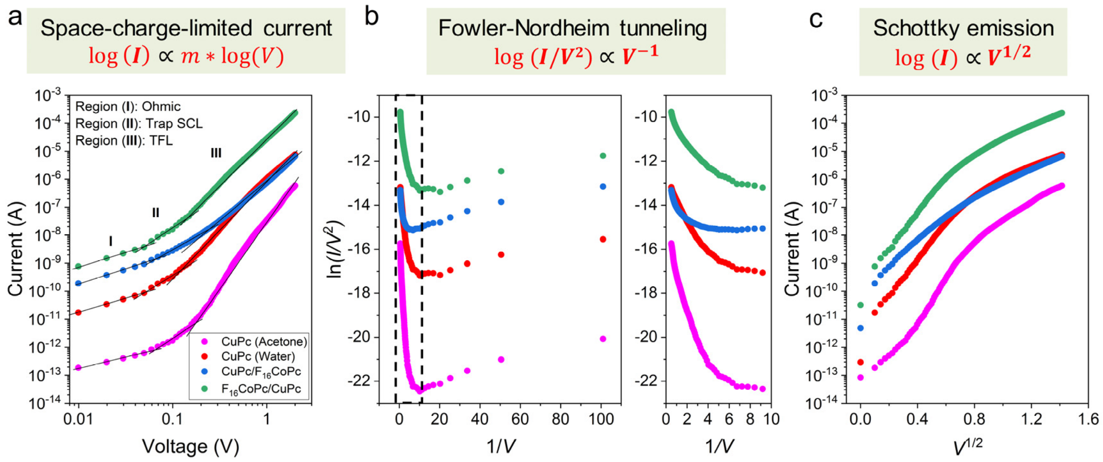

Supplementary Figure 10. Conduction models fitting for the forward *I*-*V* data. a, Space-charge-limited current model (bulk-limited). **b**, Fowler-Nordheim tunneling model (contact-limited) is applied to the forward-direction current. The right panel shows the region in the dash box of the left panel. **c**, Schottky emission model(contact-limited) is applied to the forward-direction current.

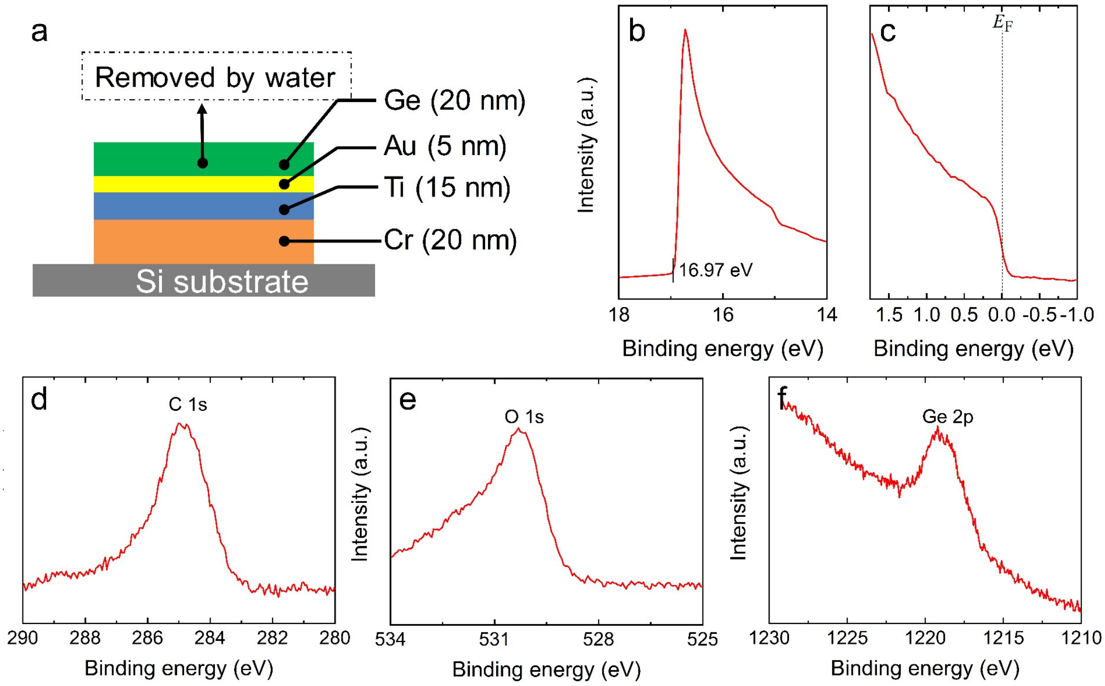

Supplementary Figure 11. **Characterization of the top tube electrode. a**, Schematic diagram of the multiple metallic layers with reverse stacking manner of the sacrificial and strain layers. Before transferring the sample to the XPS&UPS analysis chamber, Ge layer was removed by water. **c-d**, Cutoff and HOMO (or VB) regions of the sample, respectively. **d-f**, XPS spectra (Al-Kα = 1486.6 eV) corresponding O 1s, N 1s, and C 1s core levels of sample, respectively.

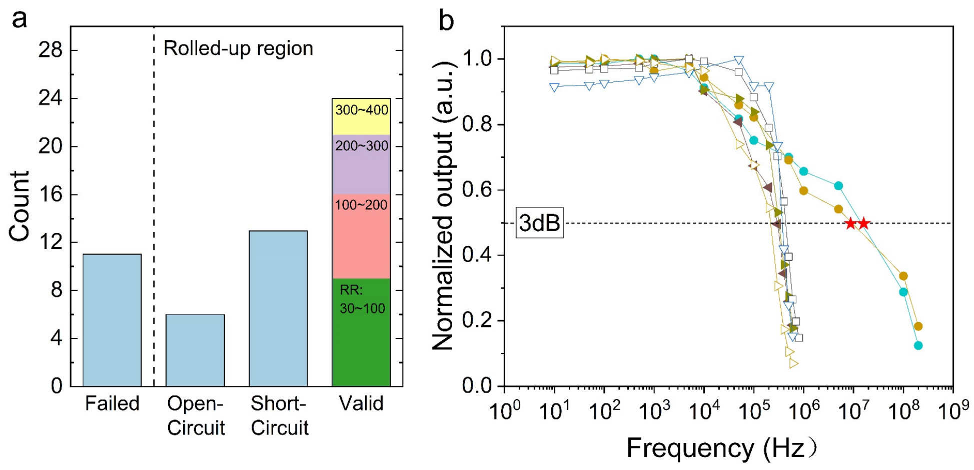

Supplementary Figure 12. Statistical analysis of the rectifier array on a chip. a, Statistics of the 54 rectifiers. RR= rectification ratio. **b**, Frequency performance of rectifiers.

Supplementary Notes

Supplementary Note 1: Thickness-related I-V performance

In this work, there exists a thickness-related tradeoff between current density and rectification ratio, as shown in Supplementary Fig. 5. With thinner molecular ensembles (such as 4.5 nm), both forward and reverse currents are high but the rectification ratio at ± 2 V is only about 15. The large leakage current could make the rectifier invalid to block the reverse current, thus losing the function of rectification. Moreover, the roughness of the top Au tube (~2.0 nm) and bottom Au finger (~1.3 nm) are in the same range of the molecule film thickness (shown in Supplementary Fig. 6). The thinner the molecular layer is, the more likely the device gets shorted or breakdown because the effective gap between the two electrodes shrinks from the peaks of the Au finger and tube electrodes, leading to a very low yield of successful rectifier devices. On the other hand, for the devices with thicker molecular layer (for instance, 15 nm), the rectification ratio is still maintained as high as about 2 orders, however the forward current is too low to act as an efficient rectifier. Therefore, we chose an 8 nm thick F_{16}CoPc/CuPc hybrid layer as the platform to investigate the electrical characteristic, which has not only high rectification ratio but also high forward current density.

Supplementary Note 2: Relation of current and mesa width

In the device fabrication, the designed mesa width (W_{design}) was defined by the lithography patterns. However, the real width (W_{real}) is generally influenced by the isotropic under-etching by HF solution during the mesa formation, as demonstrated in Supplementary Fig. 7a. During HF etching, SiO_2 is removed uniformly from all available directions, resulting in the shrunken top part of the mesa when compared to the designed size (W_{design}). As a result, the real mesa widths (W_{real}) of the designed $W_{\text{design}} = 5$ and $10\ \mu\text{m}$ are about 1.3 and 7.4 μm, respectively (shown in Supplementary Fig. 7b-c). Then the real width ratio (noted as Ratio 1) corresponding to ($W_{\text{design}} = 10\ \mu\text{m}/W_{\text{design}} = 5\ \mu\text{m}$) is 5.69 and not 2. On the other hand, as shown in Supplementary Fig. 7d-e, the average current ratio of devices based on $W_{\text{design}} = 10\ \mu\text{m}$ over $W_{\text{design}} = 5\ \mu\text{m}$ is calculated to be 5.36 (noted as Ratio 2), close to Ratio 1. Therefore, the forward current can be considered as proportional to the real mesa width.

Supplementary Note 3: Stability of the devices based on Au (finger)/F_{16}CoPc (1nm)/CuPc (7 nm)/Au (tube)

It is challenging to avoid the critical Joule heating effect of molecular-scale electronics. As shown in Supplementary Fig. 9a-b, for the devices in this work, during the 40-cycle *I-V* measurement, the rectification ratio (RR) decreased from 270 to 230, and the forward current at 2V maintained at a high level, more than 200 μA. The decrease in rectification may be caused by Joule heating which might increase the currents. However, the devices still work under such high current density due to the good thermal stability of phthalocyanine materials. On the other hand, it is found that both the forward and reverse currents increased slightly after being stored in air for 420 days without any protection, while the rectification ratio decreased a little, as shown in Supplementary Fig. 9c. This may be ascribed to the oxygen doping[1] and/or further contamination happening to the device[2]. It seems that once the device is formed, the influence of further contamination is not evident enough to dramatically change the device's performance. Our experiments also prove that there is a relatively safe current density region, above which the device tends to suffer burnout due to the excessively accumulated heat. As shown in Supplementary Fig. 9d-e, when the current density is as high as ca. 1200 A cm^{-2} at 2V (taking the contact area as 74 μm^2), the device suddenly broke down during the multiple bias scanning from positive to negative voltage. As a result, the device lost its rectifying function. Furthermore, Joule heating can also destroy the rectifying devices during the frequency measurement, as shown in Supplementary Fig. 9f.

Supplementary Note 4: Corresponding mathematic models of the three transport regimes

At low voltage, the thermally generated carrier concentration exceeds the injected concentration, and the current density is given by a form of Ohm's law[3]

$$J_{\text{Ohm}} = qn_0\mu\frac{V}{d} \qquad \text{Supplementary Equation (1)}$$

where q is the electronic charge, n_0 is the thermally generated carrier concentration (holes in the case of most phthalocyanines which are generally p type), μ is the mobility (holes of p-type phthalocyanines), V is the applied voltage and d is the film thickness.

At higher voltage, the injected carrier concentration exceeds that of the thermally generated, space-charge limit current becomes dominant. However, the existence of traps within imperfect extrinsic materials has the effect of immobilizing a large proportion of the injected carriers. If the traps are shallow and located at a discrete energy above the valence band edge of p-type CuPc, then the transport is trap-limited SCLC, given by[3]

$$J_{\text{SCLC(trap)}} = \frac{9}{8}\mu\varepsilon\theta\frac{V^2}{d^3} \qquad \text{Supplementary Equation (2)}$$

where ε is the permittivity of the semiconductor, and θ is the ratio of free to trapped carries. If the shallow traps are fulfilled, then deep traps dominate. Deep traps are close to the middle of band gap, and the transport follows trap-filled limit conduction before the fill of the traps, given by[4]

$$J_{\text{TFL}} = n_0\mu q^{1-l}\left(\frac{\varepsilon l}{H(l+1)}\right)^{l}\left(\frac{2l+1}{l+1}\right)^{l+1}\frac{V^{l+1}}{d^{2l+1}} \qquad \text{Supplementary Equation (3)}$$

where H is the trap density. This expression predicts a power-law dependence of J on V with the exponent $m = l + 1$. In theory, when the number of injected carriers reaches a maximum under the strong voltage where the traps are filled, J turns to scale quadratically with

V (i.e., $m = 2$), corresponding to trap-free SCLC. However, the applied voltage of our device below 5 V, the traps are not completed filled.

Supplementary Note 5: Transport mechanism from Au finger to molecular ensembles (bulk-limited vs. contact-limited)

The CuPc/Au (tube) interface formed by the robust mechanical contact has the ability to block the hole injection from the Au tube into the molecular layer, however, the hole crossing from the molecular layer to the Au tube is not blocked. Hence, this interface is mainly responsible for the function of rectification. This is demonstrated by the I-V characteristics (Figure 4a in the manuscript) and the energy barrier between the Au tube and the CuPc plays the major role (Supplementary Fig. 3). In other words, under forward bias condition the charge transport (from Au finger to Au tube electrode) is relatively not limited when compared to the reverse bias condition (from Au tube to Au finger electrode).To further clarify the charge transport process in forward direction, apart from the SCLC model[4], the forward-direction currents are also fitted with two most possible contact-limited conduction models (i.e., Fowler-Nordheim tunneling and Schottky emission[5]), as shown in Supplementary Fig. 10. As we can see, only SCLC model fits well, which exhibits the typical three transport regions with noticeable different slopes (shown in Supplementary Fig. 10a): Ohmic transport ($m \approx 1$, Regime I), shallow trap-limited SCLC (m≈ 2, Regime II), and deep trap-filled limit conduction ($m > 2$, Regime III)[6, 7]. This indicates, the current under forward bias complies with the trap-controlled SCLC mechanism, implying that the forward-direction transport is most likely to be bulk-limited, although the thickness of the molecular layer is only a few nanometers. Furthermore, the high-density traps (about 1.58×10^{18} cm^{-3} for water-treated CuPc) makes bulk-limited conduction possible to happen because of its high bulk resistance.

Supplementary Note 6: Statistical analysis of the rectifier array on chip

The construction of our rectifiers is based on the rolled-up nanomembrane which contacts the ultrathin molecular ensembles from the top, thus providing a damage-free and self-adjusted electrode. Any defects in sacrificial layer or strain layer could lead to the failure of rolling. For a 9×6 device array, the yield of initial devices with successful rolled-up contacts is about 80%, as shown in Supplementary Fig. 12a. Among the rolled-up devices, some are open circuit due to the disconnection of the rolled-up tubes from the circuits on the substrate, and some are short circuit. As mentioned before, the film thickness of the F_{16}CoPc/CuPc hybrid layer in the demonstrating devices is about 8 nm (shown in Figure 3d in the manuscript). However, the roughness of the top Au tube, F_{16}CoPc/CuPc hybrid layer and bottom Au finger are approximately 2.0, 2.3 and 1.3 nm (shown in Supplementary Fig. 6b-g), respectively, which are in the same range with the organic layer thickness. This could shrink the effective gap between the two electrodes sandwiching F_{16}CoPc/CuPc ultrathin layer (shown in Supplementary Fig. 6h), resulting in the short-circuit or easy breakdown. At last, about 44% (24 devices) can be obtained as successful diodes (shown in Supplementary Fig. 12a) and 13% (7 devices) possess evident rectification at frequencies larger than 100 kHz (Supplementary Fig. 12b). For the surviving rectifiers, as shown in Supplementary Fig. 12b the devices achieve a maximum 3db frequency of more than 10 MHz and an average 3dB frequency of 3.81 MHz.

Supplementary References

1. Anthopoulos T., Shafai T. Oxygen induced p-doping of α-nickel phthalocyanine vacuum sublimed films: Implication for its use in organic photovoltaics. *Appl. Phys. Lett.* **82**, 1628-1630 (2003).

2. Grobosch, M. & Knupfer, M. Charge-injection barriers at realistic metal/organic interfaces: metals become faceless. *Adv. Mater.* **19**, 754-756 (2007).

3. Chiu, F. C. Electrical characterization and current transportation in metal/Dy_2O_3/Si structure. *J. Appl. Phys.* **102**, 044116 (2007).

4. Xu, G. et al. Bulk-like electrical properties induced by contact-limited charge transport in organic diodes: revised space charge limited current. *Adv. Electron. Mater.* **4**, 1700493 (2018).

5. Chiu, F. C. A review on conduction mechanisms in dielectric films. *Adv. Mater. Sci. Eng.* **2014**, 1-18 (2014).

6. Montero, José M. et al. Trap-limited mobility in space-charge limited current in organic layers. *Org. Electron.* **10**, 305-312 (2009).

7. Samanta S. et al. Understanding of multi-level resistive switching mechanism in GeO_x through redox reaction in H_2O_2/sarcosine prostate cancer biomarker detection. *Sci. Rep.* **7**, 1-12 (2017).
